# COVID-19: potential therapeutics for pediatric patients

**DOI:** 10.1007/s43440-021-00316-1

**Published:** 2021-08-30

**Authors:** Nour K. Younis, Rana O. Zareef, Ghina Fakhri, Fadi Bitar, Ali H. Eid, Mariam Arabi

**Affiliations:** 1 Faculty of Medicine, American University of Beirut Medical Center, Riad El Solh, 11-0236, Beirut, 1107 2020 Lebanon; 2 Department of Pediatrics, Division of Pediatric Cardiology, American University of Beirut Medical Center, Riad El Solh, 11-0236, Beirut, 1107 2020 Lebanon; 3 Department of Basic Medical Sciences, College of Medicine, QU Health, Qatar University, 2713, Doha, Qatar; 4 Biomedical and Pharmaceutical Research Unit, QU Health, Qatar University, 2713, Doha, Qatar

**Keywords:** COVID-19, Pediatric patients, SARS-CoV-2, Therapeutics

## Abstract

The global spread of COVID-19 has imparted significant economic, medical, and social burdens. Like adults, children are affected by this pandemic. However, milder clinical symptoms are often experienced by them. Only a minimal proportion of the affected patients may develop severe and complicated COVID-19. Supportive treatment is recommended in all patients. Antiviral and immunomodulatory medications are spared for hospitalized children with respiratory distress or severe to critical disease. Up till now, remdesivir is the only USFDA-approved anti-COVID-19 medication indicated in the majority of symptomatic patients with moderate to severe disease. Dexamethasone is solely recommended in patients with respiratory distress maintained on oxygen or ventilatory support. The use of these medications in pediatric patients is founded on evidence deriving from adult studies. No randomized controlled trials (RCTs) involving pediatric COVID-19 patients have assessed these medications’ efficacy and safety, among others. Similarly, three novel monoclonal anti-SARS-CoV-2 spike protein antibodies, bamlanivimab, casirivimab and imdevimab, have been recently authorized by the USFDA. Nonetheless, their efficacy has not been demonstrated by multiple RCTs. In this review, we aim to dissect the various potential therapeutics used in children with COVID-19. We aspire to provide a comprehensive review of the available evidence and display the mechanisms of action and the pharmacokinetic properties of the studied therapeutics. Our review offers an efficient and practical guide for treating children with COVID-19.

## Introduction

Toward the end of 2019, a few atypical lower respiratory illness cases were witnessed in Wuhan, China [1–5]. The exact etiology of this viral-like novel respiratory illness remained unclear until the 7th of January 2020. At that time, a novel coronavirus, named severe acute respiratory syndrome coronavirus 2 (SARS-CoV-2), was extracted from patients’ respiratory tract samples [1, 2]. The disease of SARS-CoV-2, namely coronavirus disease 2019 (COVID-19), was later recognized as a global pandemic on the 11th of March, 2020, by the World Health Organization (WHO) [6, 7]. This pandemic has, since, imposed significant morbidity and mortality rates and has led to overwhelmed and weakened medical systems in all nations. As of June 23, 2021, COVID-19 has afflicted more than 178 million individuals and caused 3.88 million deaths worldwide [8].

Since the beginning of the pandemic, an effective treatment for COVID-19 has been sought. Despite the unprecedented race to find a cure, most efforts have been directed toward repurposing well-studied and -recognized medications [9, 10]. Of these medications, we list azithromycin, dexamethasone, favipiravir, hydroxychloroquine, oseltamivir, remdesivir, and ivermectin [11–14]. Immunomodulators, such as tocilizumab, and anti-HIV medicines, such as lopinavir and ritonavir, have been similarly investigated [11–14]. Additionally, many hospitalized patients with severe and complicated COVID-19 have received convalescent plasma of compatible recovered patients [11, 15–18]. The role of vitamins and minerals, including vitamin C, vitamin D, and zinc, in treating and preventing COVID-19 has been additionally examined [19–21].

Despite the above-mentioned numerous attempts and investigations, the US Food and Drug Administration (USFDA) has been very cautious and meticulous in selecting the appropriate treatment for COVID-19. In May 2020, USFDA authorized remdesivir only in patients with severe and critical diseases [22]. Toward the end of August 2020, the use of remdesivir was approved for all hospitalized patients regardless of the severity of the disease [22]. After that, on October 22 of this year, USFDA has approved the use of remdesivir in all hospitalized patients aged at least 12 years who weigh more than 40 kg [23]. Nonetheless, supportive measures, geared toward symptom control, have continued to be the core first-line management of COVID-19 in non-hospitalized patients with mild to moderate disease, as discussed below. Hydration and fever control with antipyretics represent the standard of care (SOC) offered to all patients.

This review discusses the studied potential therapeutics that can be used in treating children with COVID-19. It provides a comprehensive and updated review of the current evidence. This review reports the most updated highest-quality evidence that derives from American and European multicentered clinical trials and guidelines.

## Methods

This review intends to scrutinize the COVID-19 therapeutics that can be offered to pediatric patients. It aims also to suggest an effective treatment algorithm and discuss the accepted dosages of the potential medications and their side effects. It involves a thorough search of the recently published literature and includes studies published in the databases of Cochrane, Embase, Medline, and PubMed. We searched for the following MeSH terms and keywords: COVID-19, coronavirus disease 2019, therapeutics, therapy, treatment, azithromycin, chloroquine/hydroxychloroquine, dexamethasone, ivermectin, lopinavir/ritonavir, oseltamivir, remdesivir, tocilizumab, vitamin, and minerals. We limited our search to clinical trials, meta-analysis, and systematic reviews. We reviewed all relevant studies and inspected closely for studies involving pediatric patients. Similarly, we examined the international guidelines issued by USFDA and EMA.

## Potential therapeutics for children with COVID-19

Numerous medications have been considered as potential therapeutics for COVID-19 and investigated in pre-clinical and clinical studies. These medicines are well-known and -studied treatments commonly employed in the management of autoimmune diseases and viral, bacterial, and parasitic infections. Because of their well-established antimicrobial and anti-inflammatory properties, clinicians and researchers have endorsed the use of these therapeutic options. This use is also supported by the immunomodulatory effects exerted by some of these medicines.

The clinical course of COVID-19 in pediatric patients is mild in almost all cases [24–26]. However, severe and critical illness can affect a minor percentage of infected patients. Serious complications, such as severe pneumonia, acute respiratory distress syndrome (ARDS), multisystem inflammatory syndrome in children (MIS-C), and sepsis, can occur [25, 27–29].

Treatment of COVID-19 pediatric patients entails supportive and symptomatic management. It includes hydration with intravenous and/or oral fluids, fever reduction with antipyretics, and oxygen supplementation. The use of remdesivir and the off-label use of additional antiviral medications and immunomodulators are spared for hospitalized patients with respiratory distress and complicated disease [26, 30].

In April 2020, the American Pediatric Infectious Diseases Society endorsed therapeutic guidance provided by a group of American infectious disease specialists and pharmacists [31]. In this guidance, they recommended adopting a personalized therapeutic plan that depends on the patient’s clinical status and medical history. They endorsed the use of remdesivir in children (aged at least 12 years) with severe and critical diseases [31]. Similarly, they suggested using hydroxychloroquine (HCQ) as second-line treatment in this population (children aged 12 years and above) and as first-line treatment in younger children aged 12 years and less. The use of lopinavir and ritonavir was not approved by all the participants. Similarly, they advised against the addition of azithromycin to HCQ, and ribavirin to lopinavir/ritonavir [31].

At the end of September 2020, the Italian Society of Infectious Pediatric Diseases suggested using remdesivir in pediatric patients with severe or critical COVID-19 in whom renal and liver functions are normal [32]. HCQ and lopinavir/ritonavir should be only considered if remdesivir is contraindicated or unavailable. Similarly, immunomodulatory therapies, such as anakinra, dexamethasone, and tocilizumab, should be considered in patients with ARDS, MIS-C, prolonged disease course, or enhanced elevation in inflammatory markers [32].

Furthermore, according to the most recent treatment guideline issued by the Children’s Hospital of King’s Daughter (CHKD) in southeastern Virginia, supportive care must be offered to all patients [33]. Dexamethasone should be added in patients receiving respiratory support including oxygen and assisted ventilation. Remdesivir, on the other hand, should be reserved for hospitalized COVID-19 patients who are on invasive ventilators. Its use is contraindicated in patients with (1) multiorgan dysfunction, (2) vasopressor support, (3) markedly elevated alanine aminotransferase (ALT), (4) severe renal impairment (GFR < 30 ml/min and dialysis), or (5) concurrent administration of other antivirals [33]. Tocilizumab and anakinra must be considered when remdesivir is contraindicated or inaccessible. These medications are also indicated in patients at high risk for developing complicated disease reflected by severe worsening of respiratory function despite mechanical ventilation, and markedly elevated inflammatory markers (ferritin, Il-6, LDH, and D-dimer) [33]. They are also indicated in patients with severe MIS-C. Intravenous immunoglobulin (IVIG), aspirin, and anticoagulants are indicated in patients with MIS-C regardless of severity [33].

The use of other therapeutics, including azithromycin, ivermectin, and oseltamivir, among others, has been likely explored [34, 35]. Herein, we provide a review of the latest highest-quality evidence that discusses the anti-COVID-19 properties and the efficacy of the following medications: azithromycin, chloroquine/hydroxychloroquine, dexamethasone, ivermectin, lopinavir/ritonavir, oseltamivir, remdesivir, and tocilizumab. We also describe the therapeutic role of the novel monoclonal anti-SARS-CoV-2 spike protein antibodies recently authorized by the USFDA. The role of vitamin and mineral supplements in treating COVID-19 is equally discussed. This review aims to convey a brief description of the mechanism of action and the pharmacokinetic properties of the aforementioned medications. Due to the lack of randomized controlled trials (RCTs) involving pediatric patients, most of the below evidence is derived from high-quality adult RCTs.

### Chloroquine/hydroxychloroquine

#### Mechanism of action

**Fig. 1:**
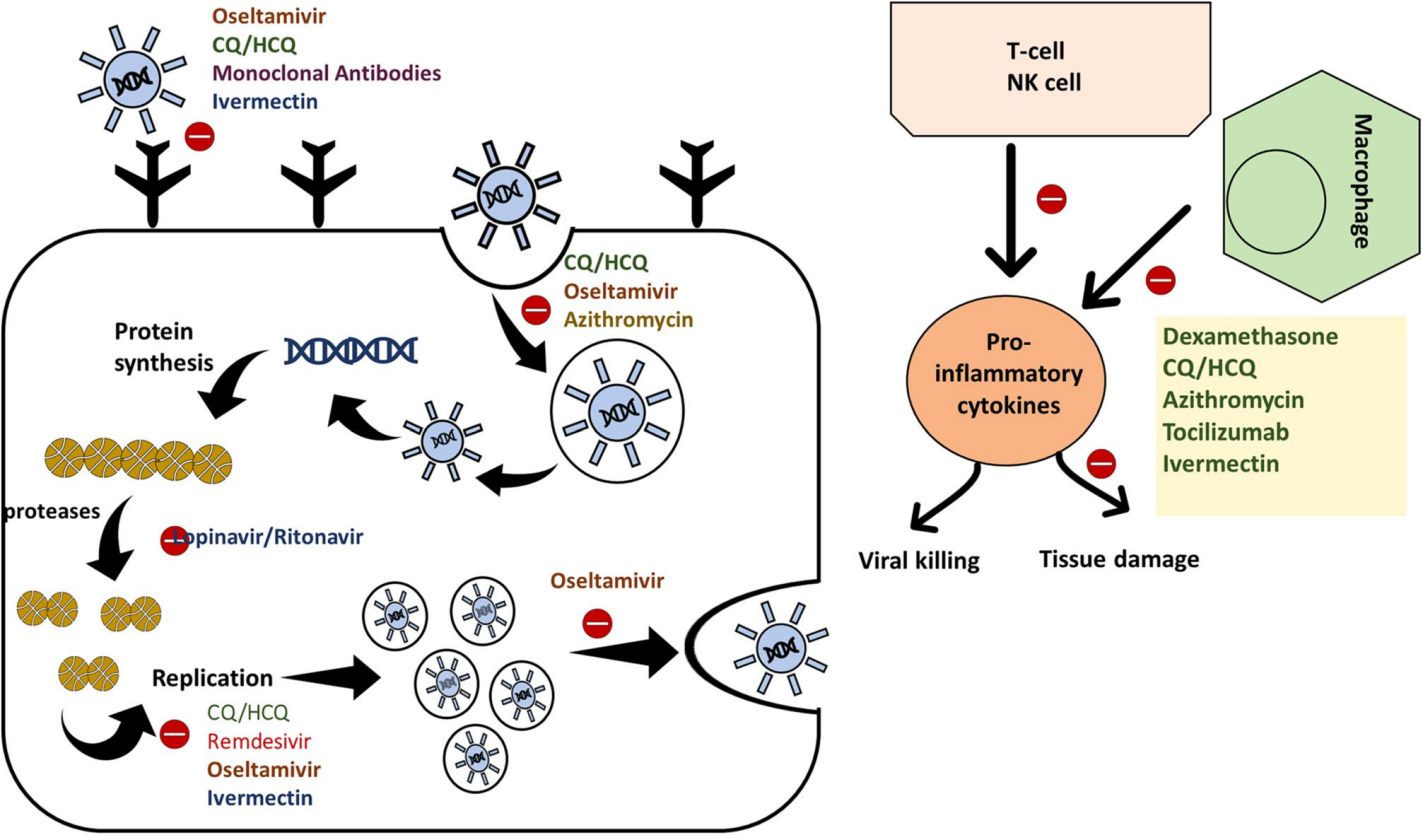
The potential therapeutics of COVID-19 and their suggested mechanisms of action. Chloroquine/hydroxychloroquine (CQ/HCQ) is known for its anti-inflammatory and antimicrobial effects. They inhibit viral fusion, endocytosis, and intracellular replication. Similarly, they modulate the inflammatory response mounted against the virus by attenuating the excessive uncontrolled release of pro-inflammatory cytokines. Oseltamivir is a selective inhibitor of the neuraminidase enzymes of the influenza virus. These enzymes are involved in various viral processes including viral entry, replication, packaging, and release. Hence, their use in SARS-CoV-2 infection may thwart the cellular processing of this virus at different levels. Remdesivir is a potent inhibitor of viral replication. It exerts its effect through the selective inhibition of the RNA-dependent RNA polymerase. Remdesivir, unlike CQ/HCQ, oseltamivir, and ivermectin, is a direct inhibitor of RNA-dependent RNA polymerase. CQ/HCQ, oseltamivir, and ivermectin hinder indirectly the replication through a cascade of events. On the contrary, dexamethasone, azithromycin, and tocilizumab are recognized for their anti-inflammatory effects. They control the inflammatory response mounted against the infection and hamper the progression to uncontrolled hyperinflammation and tissue destruction. These medications may alleviate the cytokine storm syndrome that may be associated with severe and complicated SARS-CoV-2 infections. Hence, they attenuate the tissue damage that may accompany viral killing. Similarly, the newly synthesized anti-SARS-CoV-2 spike protein antibodies are inhibitors of viral fusion and internalization. Viral endocytosis is also impeded by azithromycin. Moreover, ivermectin seems to inhibit viral uptake and replication as well as pro-inflammatory cytokines production

The synthetic derivatives of quinine, chloroquine (CQ) and hydroxychloroquine (HCQ), were formerly employed in treating malaria, autoimmune, cutaneous, and rheumatic diseases [36]. Similarly, CQ and HCQ were effective against various bacterial and viral infections including *Coxiella burnetii*, *Tropheryma whipplei, Ebola virus, Hepatitis C, and chikungunya* [36]*.* Their clinical uses are derived from their anti-inflammatory, immunomodulatory, and antimicrobial properties. CQ/HCQ inhibits viral entry and intracellular replication (see Fig. 1). They exert their antiviral effects via (1) the inhibition of sialic acid synthesis, (2) the binding to sialic acid and gangliosides, (3) the impairment of viral cellular interaction, and (4) the interference with intracellular pathways that are key for viral replications such as p38 mitogen-activated protein kinase (MAPK) pathway [36]. Similarly, they boost the dendritic cells’ activity which, in turn, enhances the activity of cytotoxic T cells. They are known to attenuate the inflammatory response mounted against pathogens and autoimmune triggers. This effect is achieved via the downregulation of the release of pro-inflammatory cytokines (such as Il1-α, TNF-α, and Il6) that can result in hyperinflammation and tissue destruction [36].

#### Pharmacology

The elimination half-life of HCQ is estimated at 40–50 days [37]. The bioavailability of oral CQ and HCQ is estimated at 70–80%. Hence, their use in the oral formulation can achieve adequate therapeutic concentrations in most patients [37]. CQ and HCQ display moderate binding to plasma proteins and have high volumes of distribution [36–38]. No dosage adjustment is indicated in patients with liver and kidney diseases. However, the drugs are metabolized by the liver and excreted in urine and feces [38]. Hence, caution should be applied when treating patients with liver or kidney dysfunction.

#### Clinical evidence

A retrospective cohort study involving 582 pediatric patients admitted to 82 European hospitals reported that hydroxychloroquine, remdesivir, and lopinavir–ritonavir were the most-used medications [39]. However, up till now, no RCTs have assessed the efficacy of these medications, among others, in treating pediatric COVID-19 patients. The currently available evidence is solely derived from adult-targeted studies.

Based on the latest evidence deriving from high-quality RCTs, hydroxychloroquine has been found non-superior to standard care in the treatment of adult COVID-19 patients [40, 41]. The SOLIDARITY trial, conducted by the WHO, has the largest number of enrolled patients (around 12,000 patients) treated in more than 500 hospitals globally. As per the preliminary results of this trial, little or no benefit can be induced by the addition of hydroxychloroquine to the standard care of COVID-19 patients. Similarly, in this study, remdesivir and lopinavir–ritonavir were not associated with a significant improvement in mortality and in-hospital clinical status [40].

Congruently, the RECOVERY collaborative group has assessed the efficacy of hydroxychloroquine and usual care in a cohort of 4716 patients [41]. One thousand five hundred and sixty-one patients were randomly assigned to the hydroxychloroquine-treated group. The rest of the patients were only provided with supportive care. No improvement in 28-day mortality and overall clinical outcomes was caused by the addition of HCQ [41]. Moreover, a higher incidence of mortality and transition to mechanical ventilation was encountered in the HCQ-treated group. A mildly increased incidence of cardiac-related deaths was witnessed in this group as well [41].

The studies mentioned above represent the largest studies with the highest quality of evidence. They are superior to the formerly published observational studies that supported the use of HCQ in treating COVID-19 [36, 42–45]. These former studies are limited by the small number of enrolled patients, the lack of control and randomization, and the known inherent biases of retrospective observational studies. In short, the use of HCQ cannot be warranted based on the currently available data. Hence, further RCTs are needed to elucidate the exact role of HCQ and endorse alternative safe and effective medications.

### Dexamethasone

#### Mechanism of action

Dexamethasone is a corticosteroid with potent anti-inflammatory and immunomodulatory activities. It downregulates the immune response mounted against endogenous autoimmune insults, such as auto-antibodies targeted against self-antigens, and exogenous triggers such as SARS-CoV-2. It exerts this effect by inhibiting neutrophils’ migration to inflammatory sites, and inducing apoptosis of basophils, eosinophils, lymphocytes, and monocytes [46]. Dexamethasone is also known to inhibit lymphocytes proliferation. Derived from its immunosuppressive effects, the use of dexamethasone is associated with a diminished expression and production of pro-inflammatory cytokines and an increased expression and production of anti-inflammatory cytokines [47–49]. Similarly, dexamethasone is employed in treating numerous pulmonary diseases such as asthma, chronic bronchitis and emphysema [50]. It reduces pulmonary inflammation resulting in improved respiratory functions [50]. Hence, it is postulated that the addition of dexamethasone to standard care is warranted in COVID-19 patients with respiratory distress and severe disease.

#### Pharmacology

The half-life of dexamethasone is estimated at 4.1 to 5.4 h [51]. It has a good oral bioavailability estimated at around 70–80% [51, 52]. Hence, it can be administered orally or intravenously. Dexamethasone exhibits extensive plasma protein binding and its volume of distribution may be as high as 99 L [51]. The liver metabolizes it into more water-soluble metabolites excreted by the kidneys [51]. Hence, careful dosing and monitoring of side effects are mandatory when dealing with hepatic and renal diseases.

#### Clinical evidence

Owing to its favored anti-inflammatory and respiratory effects, the use of dexamethasone in patients with COVID-19 has gained much attention. This use has been examined by several groups of medical clinicians and researchers. On the 17th of July 2020, the RECOVERY collaborative group has released a preliminary report discussing dexamethasone’s efficacy in hospitalized COVID-19 patients [53]. They conducted an RCT enrolling 6425 patients. 2104 and 4321 patients were randomly assigned to the dexamethasone and the standard care groups, respectively. A daily dexamethasone dose of 6 mg was administered to the treated group for up to 10 days [53].

Overall, the mortality rate was significantly lower in the dexamethasone group. However, clinical outcomes and mortality were influenced by the baseline clinical status of the patients. Stratified statistical analysis as per the patients’ need for respiratory support ((1) no support vs (2) oxygen and/or mechanical ventilation) showed varied clinical outcomes among dexamethasone-treated patients [53]. A significant improvement in mortality and clinical outcomes was noted in patients already receiving respiratory support. Yet, worsened clinical outcomes and increased mortality were encountered in the dexamethasone-treated patients who initially required no respiratory support [53]. Hence, the use of dexamethasone was only endorsed in patients with severe disease requiring supplemental oxygen and ventilation support.

This randomized controlled trial conveys preliminary evidence. However, because of its large sample size and feasible design, it is considered a breakthrough study that delivers high-quality pivotal evidence. The study is limited by the lack of blinding and the advanced age of the enrolled patients. No pediatric patients were enrolled in this trial.

Congruently, Tomazini et al. reported promising outcomes. His team performed a multicenter, controlled, randomized, controlled trial involving 299 COVID-19 patients admitted to the intensive care unit [54]. They administered intravenous dexamethasone to 151 patients at a dose of 20 mg once daily for 5 days, followed by a daily dose of 10 mg until discharge. The addition of dexamethasone to standard care was linked to significantly improved respiratory outcomes and also to a reduced need for respiratory support [54]. This study has several limitations. The effect of dexamethasone on COVID-19 mortality was not assessed in this trial due to the limited follow-up duration. The trial was terminated after the favorable results of the RECOVERY trial were released. This study is also limited by its participants’ advanced age and the lack of participants aged below 18 years.

In short, favorable outcomes are associated with the use of dexamethasone in patients with severe and critical COVID-19 requiring respiratory support. Nevertheless, no evidence has been derived from pediatric studies, and no pediatric patients have been included in any of the conducted adult studies. High-quality pediatric clinical trials and observational studies are needed to validate the efficacy of this medication in children with COVID-19.

### Ivermectin

#### Mechanism of action

Ivermectin is an FDA-approved broad-spectrum antiparasitic agent applied in clinical practice since 1987 [55]. In addition to its well-known antiparasitic activity, ivermectin exhibits antineoplastic and antiviral properties [56, 57]. Its anti-viral activity against RNA viruses, such as flaviviruses, is mediated by inhibiting the nuclear localization of viral proteins. Moreover, ivermectin’s activity against HIV and dengue viruses, is prompted by the inhibition of importin α/β-mediated nuclear transport, a vital process in viral life cycle [58]. Ivermectin impedes the replication of DNA viruses, like pseudorabies, through the disruption of the nuclear localization of the protein UL42, a key mediator of DNA synthesis [59]. In the setting of SARS-CoV-2 infection, ivermectin seems to be an inhibitor of both viral entrance and replication. It disrupts the interaction between spike receptor binding domain and ACE2 receptor, and prevents subsequently SARS-CoV-2 cellular entry [60]. Ivermectin thwarts as well viral replication via its binding to RNA-dependent RNA polymerase (RdRp) [61].

The anti-inflammatory properties of ivermectin has been also documented. Ivermectin is a potent suppressor of the inflammatory response prompted by bacterial LPS. This anti-inflammatory effect is imparted by attenuating the production of pro-inflammatory cytokines such as TNF-α, IL-1ß, and IL-6 [62]. Similarly, in mouse asthma model stimulated with ovalbumin, ivermectin significantly suppresses airway inflammation and hyperactivity via the inhibition of cytokines production and immune cells’ recruitment [63].

#### Pharmacology

Ivermectin belongs to a group of 16-membered macrocytic lactone compounds called avermectins [64]. The pharmacology of ivermectin is well studied. Ivermectin is a highly fat-soluble agent with a large distribution volume of 46.9 L. In the human body, around 93% of the ivermectin molecules are bound to plasma proteins [65]. Ivermectin’s extensive binding to plasma proteins is a crucial limiter of its cellular uptake. No evidence of central nervous system penetration in humans was recorded [55]. Ivermectin displays a peak plasma level 5 h after an adequate oral dose, and has a half-life of 36 h and a clearance of 1.2 L/h [64]. The plasma level of its metabolites is maintained for a longer time due to enterohepatic recycling [64]. Ivermectin is extensively metabolized in the liver microsomes by cytochrome P450, and is excreted mainly by feces (98%) [64]. The usual dose used for antiparasitic purposes ranges from 150 to 200 µg/kg, but a higher dose of up to 400 µg/kg is used for treatment of lymphatic filariasis [64, 66].

#### Clinical evidence

Ivermectin clinical efficacy against SARS-CoV-2 has been evaluated in several small sample-sized RCTs and observational studies. A randomized, double-blinded, placebo-controlled trial was performed in Bangladesh to assess the efficacy of ivermectin alone or in combination with doxycycline on viral clearance as well as its safety. The study included 72 hospitalized individuals diagnosed by positive PCR tests. No significant difference was found among the different study arms in terms of clinical symptoms. Earlier viral clearance was noted in the ivermectin group compared to placebo, but not in the ivermectin/doxycycline combination group [67]. Another randomized double-blinded placebo-controlled study was carried out in one center in India to evaluate the efficacy of ivermectin against COVID-19. The study included 112 individuals aged 18 years and above. Patients either received 2 mg of ivermectin on days 1 and 2 or placebo tablets. The ivermectin-treated group had significantly a higher incidence of alive discharged participants (100%) compared to the placebo group (93%) [68].

The ICON study is a published retrospective cohort multicenter study in South Florida that investigated the effect of ivermectin treatment on overall COVID-19 in-hospital-mortality, severe pulmonary manifestations mortality, extubation rate, and hospitalization length. The study included 280 COVID-19-confirmed patients, of which 173 received ivermectin. The study showed that ivermectin is associated with a lower overall mortality rate (15 vs. 25%), and a lesser severe pulmonary manifestations associated mortality (38.8 vs 80%). Nonetheless, there was no significant improvement in extubation rates or hospitalization length [69].

In short, evidence supporting the anti-COVID-19 activity of ivermectin is still controversial. Due to the lack of large-scale randomized clinical trials, the role of ivermectin in treating COVID-19 remains to be proven. Also, studies on pediatric patients are lacking. Hence, its clinical use in pediatric population cannot be founded on the current evidence.

### Lopinavir/ritonavir

#### Mechanism of action

Lopinavir and ritonavir are HIV-1 protease inhibitors that prevent the formation of mature and infectious HIV-1 progeny (Table 1) [70]. They are often administered in combination and merged in one pill; low-dose ritonavir is added to boost the plasma concentration of lopinavir and enhance its activity [71].

**Table 1:** Mechanism of action and efficacy of the above-mentioned potential therapeutics of COVID-19

Medication	Mechanism of action	Superior/ non-superior to SOC* Class of recommendation/ level of evidence
Hydroxychloroquine	Displays various anti-inflammatory and immunomodulatory properties Inhibits SARS-CoV-2 entry and replication Downregulates the cytokine storm syndrome mounted against SARS-CoV-2	Non-superior to SOC Class of recommendation IIb^+^ (IIa in some parts of the world) Level of evidence C^#^
Dexamethasone	Exhibits various anti-inflammatory and immunomodulatory properties Reduces pulmonary inflammation and improves respiratory function Downregulates the cytokine storm syndrome mounted against SARS-CoV-2	Superior to SOC in patients with respiratory distress maintained on oxygen or ventilatory support Class of Recommendation: **As per US NIH:** **I for** patients requiring respiratory support **III** for patients not requiring respiratory support **As per EMA:** **I** for patients requiring respiratory support **III** for patients not requiring respiratory support Level of evidence C
Ivermectin	Exerts antiviral and anti-inflammatory effects Interferes with SARS-CoV-2 cellular entry, and intracellular replication	Superior to SOC as per evidence deriving from small-sized RCTs **(However, larger higher-quality RCTs are needed to endorse further its anti-COVID-19 activity)** Class of Recommendation IIIC **(Its use is not yet endorsed by EMA and USFDA)** Level of evidence C
Lopinavir/Ritonavir	Inhibits HIV-1 Protease and prevents the formation of mature and infectious HIV-1 progeny	Non-superior to SOC Class of recommendation IIb Level of evidence C
Remdesivir	Binds to the RNA-dependent RNA polymerase and prevents the binding of endogenous nucleosides to the growing viral RNA	Superior to SOC **(However, its use was not supported by the SOLIDARITY study, conducted by WHO, as discussed in our article)** Class of recommendation I (as per USFDA and EMA) Level of evidence C
Tocilizumab	A humanized monoclonal antibody targeted against interleukin-6 (Il-6) receptor Prevents binding of Il-6 to its receptor Downregulates the cytokine storm syndrome mounted against SARS-CoV-2	Non-superior to SOC Class of recommendation IIb Level of evidence C
Azithromycin	Macrolide antibiotic with antiviral and anti-inflammatory properties	Non-superior to SOC when combined with HCQ Class of recommendation IIb Level of evidence C (**No data is available regarding its efficacy as a monotherapy**)
Oseltamivir	Selective inhibitor of neuraminidase enzyme	No RCTs have assessed its efficacy; Studies are still ongoing Class of recommendation IIb Level of evidence C
Monoclonal antibodies (bamlanivimab, casirivimab/imdevimab)	Newly synthesized anti-SARS-CoV-2 spike protein monoclonal antibodies Prevent viral attachment to human cells	Superior to SOC in non-hospitalized COVID-19 patients with mild to moderate disease requiring no respiratory support (**Their emergency use authorization by USFDA was based on interim results reported by couple of studies. Trials are still ongoing)** Class of recommendation IIa/IIb for at high-risk patients (**still unclear**) Level of evidence C

*HCQ* Hydroxychloroquine, *SOC* Standard of care, *RCTs* Randomized controlled trials, *US NIH* US National Institute of Health, *EMA* European Medicines Agency, *USFDA* US Food and Drug Administration

The Level of Evidence is C since no pediatric RCTs have been conducted. Most of the discussed evidence is extrapolated from adult RCTs and experts’ opinions. (*as per the latest highest-quality evidence. ^**+**^**Classes of recommendations: (I)** medication is indicated/recommended, **(IIa):** medication should be considered, **(IIb):** medication can be considered, **(III):** medication is not recommended**. **^**#**^**Levels of evidence: Level A:** evidence derived from multiple RCTs or meta-analysis**, Level B:** evidence derived from one RCT or multiple large observational studies**, Level C:** evidence derived from experts’ opinion, guidelines, and small retrospective observational studies.**)**

#### Pharmacology

The estimated half-life of a single dose of lopinavir/ritonavir is 2–3 h. It increases to 4–6 h when multiple doses are administered [70]. The exact bioavailability of oral lopinavir/ritonavir is not yet known. The main component of the lopinavir/ritonavir combination is lopinavir. Due to its lipophilicity, lopinavir is highly bound to plasma protein (almost 100%). It is metabolized by the liver CYP3A4 enzyme [70]. Ritonavir, an inhibitor of HIV protease and CYP3A4, is added to increase lopinavir’s therapeutic plasma concentration. The metabolites of lopinavir/ritonavir are eliminated in the stool and not renally excreted. Cautious dosing should be applied when administering the drug to patients with hepatic impairment [70].

#### Clinical evidence

Given that both HIV and coronaviruses are single-stranded RNA viruses, the combination therapy of lopinavir and ritonavir was previously employed in treating patients with SARS-CoV and Middle-East-respiratory-syndrome-coronavirus (MERS-CoV) [72–74]. Similarly, the anti-SARS-CoV-2 activity of this combination was assessed in in-vitro studies. In one study, tolerable doses of lopinavir/ritonavir (7/1.75 μg/mL) were applied to Vero cells infected with SARS-CoV-2 [75]. A significant reduction in viral cytopathy and replication was noted in the lopinavir/ritonavir-treated cells compared to the non-treated cells [75]. Driven by these promising results and by the anti-SARS-CoV and -MERS-CoV activities of lopinavir/ritonavir, several clinical trials have explored the efficacy of this combination in COVID-19 patients.

In a recent study, published on the 24th of October 2020, lopinavir/ritonavir was compared to standard supportive care in a cohort of 5040 patients. Lopinavir/ritonavir (400/100 mg) was administered, in addition to standard supportive care, to 1616 hospitalized COVID-19 patients [76]. No significant improvement in mortality rate was noted in the lopinavir–ritonavir-treated group as compared to the control group. Similarly, the rate of transition to mechanical ventilation was comparable between the two groups [76]. This study conveys the latest highest-quality evidence owing to its large sample size and appropriate design. Adequate randomization and control were applied. However, the study is subjected to a few limitations. No pediatric patients were enrolled in this study, and only a limited number of critically ill intubated patients were enrolled. Furthermore, only oral preparations of the combination therapy were available for this study. Hence, the use of these preparations was limited in patients with swallowing difficulties or gastrointestinal intolerance.

Moreover, a combination of lopinavir and ritonavir (400/100 mg) was not superior to standard care in hospitalized patients with severe COVID-19. In a cohort of 199 patients, 99 patients were assigned to lopinavir/ritonavir and 100 patients to standard care [77]. There was no significant difference in the time to clinical improvement and the mortality rate between the two groups. Similarly, a few days after treatment initiation, the combination therapy was halted in 13.8% of the treated patients due to side effects [77]. Just like the previous study, this study is adequately controlled and randomized. However, it is limited by (1) the limited number of patients, (2) the lack of pediatric patients, and (3) the existence of several confounders including the difference in the baseline characteristics of the patients enrolled, and the use of concurrent medications such as dexamethasone in some patients. Both studies were not blinded and thus are subject to observer bias.

In a separate study, lopinavir/ritonavir was administered either alone or in combination with ribavirin and interferon beta-1b to hospitalized COVID-19 patients treated in several hospitals in Hong Kong, China [78]. The triple therapy, consisting of lopinavir/ritonavir, ribavirin, and interferon beta-1b, was found superior to the monotherapy of lopinavir in 127 patients (86 vs 41 patients, respectively) [78]. Faster recovery and improved clinical outcomes were noted in the triple therapy group. However, no placebo or standard care control group was included in this study. Similarly, no pediatric patients were enrolled and only those with mild to moderate disease were included. Overall, based on the aforementioned evidence, we argue that a combination of lopinavir/ritonavir is ineffective in adult patients with COVID-19. Similarly, this preparation has not yet been studied in high-quality pediatric RCTs. Hence, its use in pediatric patients cannot be supported by the available evidence.

### Remdesivir

#### Mechanism of action

Remdesivir is a novel medication employed in treating numerous RNA-based viral infections. It was initially fostered for the treatment of Ebola virus in 2014 [79, 80]. The development of remdesivir was based on previously discovered and engineered nucleoside analogs that are effective in treating RNA viruses [79–81]. Remdesivir, or GS-5734, is a monophosphoramidate adenine nucleoside analog. It binds to the RNA-dependent RNA polymerase and prevents endogenous nucleosides’ binding to the growing viral RNA. Hence, it inhibits intracellular replication of RNA viruses such as Ebola, SARS-CoV, MERS-CoV, and SARS-CoV-2 [79–81]. Remdesivir is considered effective against numerous coronaviruses owing to the preserved structure of the non-structural protein 12 (nsp12) of the RNA-dependent RNA polymerase. This protein is shared by various coronaviruses and acts as a target for many antiviral medications including remdesivir [81].

Remdesivir is an inactive pro-drug that is converted by cellular enzymes into its active form: GS-443902. GS-443902 is a nucleoside triphosphate [81]. In addition to its activity against viral replication, remdesivir can escape a challenging viral mechanism imposed by coronaviruses. These viruses are recognized by their ability to express a distinctive exoribonuclease (ExoN) and thus to resist many antiviral therapeutics. The efficacy of remdesivir is partly affected by the presence of this enzyme. However, its efficacy and potency are still maintained by its ability to escape proofreading by ExoN [81].

#### Pharmacology

The half-life of remdesivir is estimated at 1 h when administered intravenously. However, the half-life of its active metabolite is around 40 h. The oral bioavailability of remdesivir is inadequate due to its extensive first-pass metabolism. Hence, remdesivir should be administered intravenously to ensure an adequate therapeutic effect [81]. The plasma protein binding of remdesivir and its active metabolite are moderate and low, respectively. Remdesivir is believed to be metabolized by plasma hydrolases and, to a lesser extent, by hepatic enzymes. Thus, its use is contraindicated in patients with severe liver disease. The renal excretion of remdesivir is minimal; however, one of its metabolites GS-441524 is moderately excreted by the kidneys [81]. Hence, the use of remdesivir is not advised in patients with impaired renal functions and particularly in those with a glomerular filtration rate below 30 ml/min. The pharmacokinetic properties of remdesivir are derived from experimental studies and adult clinical trials [81]. No studies have assessed the pharmacokinetics of remdesivir in pediatric patients.

#### Clinical evidence

The role of remdesivir in treating coronaviruses (i.e., SARS-CoV, MERS-CoV, and SARS-CoV-2) has been explored in numerous in vitro and in vivo animal studies [81]. Based these studies’ preliminary data, remdesivir was deemed effective in treating these respiratory illnesses and preventing their pervasive propagation. Congruently, early in the course of the COVID-19 pandemic, its efficacy was assessed in a few case reports and observational studies [81]. These studies were limited by the small sample size, the limited ability to assess the moderate- and long-term efficacy and safety of remdesivir, and the lack of comparison groups. Hence, the evaluation of remdesivir in high-quality controlled randomized clinical trials was needed to endorse its safety and efficacy in COVID-19 patients.

On the 8th of October 2020, Beigel et al. released the final results of their clinical trial [82]. In this trial, they compared the efficacy of remdesivir to placebo in a cohort of 1062 patients. Five hundred and forty-one patients were treated with remdesivir over 10 days; they received a loading dose of 200 mg on the first day, and then a 9-day course of 100 mg [82]. As compared to the placebo group, the time to recovery was significantly shorter in the remdesivir group. A faster improvement in clinical conditions was noted in the remdesivir group [82]. Similarly, lower mortality rates were detected among the patients treated with remdesivir after 15 and 29 days of treatment initiation, respectively. The enhanced clinical improvement was statistically significant; yet, the decrease in mortality rates was not significant. More side effects were experienced by the placebo group [82]. As compared to other randomized clinical trials, this study conveys the latest highest-quality evidence [83, 84]. It has the largest number of patients and is adequately controlled and double-blinded. Patients were randomly selected from 13 countries and 60 centers. 85% of the patients had severe COVID-19 and the average age of the participants was 58.9 years. No children were enrolled in this study. Moreover, these beneficial outcomes contradict the preliminary results reported by the SOLIDARITY trial, as mentioned previously. Hence, we argue that the use of remdesivir in children cannot be based on this study. Further clinical trials, with enrolled pediatric patients, are needed to assess the efficacy and safety of remdesivir not only in adults but also in pediatric patients.

Furthermore, Goldman et al. compared the efficacy of 5-day and 10-day courses of remdesivir in a cohort of 397 patients [85]. All patients were provided with a 200 mg loading dose on the first day of treatment and then with a daily dose of 100 mg for 4 or 9 days. After 14 days of treatment initiation, comparable clinical outcomes were detected in both groups. No significant difference was noted between the two regimens of remdesivir [85]. Hence, a 5-day course of treatment can provide a favored and cost-effective approach for COVID-19 management. Nonetheless, no children were enrolled in this study, as well.

In a recently published trial, the efficacy of a combination therapy consisting of remdesivir and a Janus kinase inhibitor, baricitinib, was compared to remdesivir plus placebo in a cohort of 1033 COVID-19 hospitalized patients [86]. Superior outcomes were achieved in the combination therapy group, particularly among patients receiving high-flow oxygen or non-invasive ventilatory support. There was no significant improvement in 28-day mortality. However, fewer side effects were encountered in the combination therapy group [86]. Collectively, these findings indicate that remdesivir anti-COVID-19 efficacy may be supplemented by additional potent immunomodulators like baricitinib.

Overall, as per the above-mentioned studies, remdesivir has a good safety profile. Most of its side effects are self-limited gastrointestinal symptoms [82, 85]. Tables 2 and 3 show the adequate doses of the discussed medications and their potential side effects, respectively.

**Table 2:** Adequate and proper dosing and duration of the potential therapeutics of COVID-19 [32, 33, 122, 132]

Medication	Dose in adults	Dose in children
Hydroxychloroquine	A first dose of 800 mg (two doses of 400 mg) on day 1, followed by a daily dose of 400 mg (200 mg twice daily) for a total of 4–7 days	Weight < 50 kg: A first daily dose of 12 mg/kg (two doses of 6 mg/kg) on day 1 followed by a daily dose of 6 mg/kg (3 mg/kg twice daily) for a total of 4–7 days Weight ≥ 50 kg: Same as adults
Dexamethasone	0.15 mg/kg once daily for up to 10 days (maximal dose 6 mg)	
Ivermectin	At least one dose of 0.2 mg/kg (a repeat dose can be given at 7 days)	Its use in children with COVID-19 is not well studied or reported
Lopinavir/Ritonavir	400/100 mg two times per day for a total of 10–14 days	Age 4 days to 1 year: 300/75 mg/m^2^ two times per day for a total of 7 days Age 1 to 18 years: 12/3 mg/kg two times per day for a total of 7 days
Remdesivir	A loading dose (LD) of 200 mg followed by a daily maintenance dose (MD) of 100 mg for a total of 5–10 days	Weight < 40 kg: A LD of 5 mg/kg followed by a daily MD of 2.5 mg/kg Weight ≥ 40 kg: Same as adults
Tocilizumab	A single dose of 8 mg/kg (maximal dose 800 mg)	Weight < 30 kg: A single dose of 12 mg/kg (maximal dose 800 mg) Weight ≥ 30 kg: A single dose of 8 mg/kg (maximal dose 800 mg)
Azithromycin	A first dose of 500 mg, followed by a daily dose of 250 mg for 4 days	A first dose of 10 mg/kg followed by a daily dose of 5 mg/kg for 4 days
Oseltamivir	75 mg twice daily for a total of 5 days (**N.B:** The duration of treatment in immunocompromised adults and children is 10 days.)	Weight 10–15 kg: 30 mg twice daily for a total of 5 days Weight 15–23 kg: 45 mg twice daily for a total of 5 days Weight 24–40 kg: 60 mg twice daily for a total of 5 days Weight ≥ 40 kg: Same as adults

**Table 3:** Safety profile of the above-mentioned potential therapeutics of COVID-19 [32, 33, 83, 103, 122, 132–140]

Medication	Potential side effects	Cautions	Contraindications
Hydroxychloroquine	Diarrhea, elevated liver enzymes, nausea, vomiting, myopathy, headache, retinopathy, cardiac arrhythmias (AV block, QT interval prolongation…etc.)	Severe renal or liver diseases Severe GI, CNS or hematologic diseases G6PD deficiency Arrhythmias	Hypersensitivity Preexisting retinopathy Pregnancy
Dexamethasone	Hypertension, Hyperglycemia	CKD Diabetes mellitus GI ulcers Heart failure Hypertension	Hypersensitivity Fungemia Avoid live and live-attenuated vaccines
Ivermectin	Gastrointestinal side effects, hypotension, cutaneous rashes, neurotoxic symptoms (confusion, seizure, and lightheadedness)	Moderate to severe hepatic dysfunction	Hypersensitivity
Lopinavir/Ritonavir	Diarrhea, elevated liver enzymes, nausea, vomiting, cardiac arrhythmia (prolonged PR/QT interval)	Mild to moderate hepatic dysfunction Dyslipidemia Diabetes mellitus	Hypersensitivity Severe hepatic dysfunction
Remdesivir	Constipation, diarrhea, nausea, vomiting, elevated liver enzymes, hypotension, anemia, thrombocytopenia, hyperglycemia, hypersensitivity	Mild to moderate hepatic and renal dysfunction	Hypersensitivity Stage 4–5 CKD Severe hepatic dysfunction
Tocilizumab	Gastrointestinal side effects, hepatitis, neutropenia, lymphopenia, anemia, hypersensitivity	Untreated latent tuberculosis (TB) Chronic Hepatitis B infection Active/Chronic liver disease Hematologic disease Avoid live and live-attenuated vaccines	Hypersensitivity Pregnancy Breastfeeding Active TB/ systemic fungal infections
Azithromycin	Gastrointestinal side effects, headache, skin rashes, cardiac arrythmias (QT interval prolongation, bradycardia)	Severe hepatic or renal disease Cardiac arrhythmias	Hypersensitivity
Oseltamivir	Gastrointestinal side effects, cough, nasal congestion, fatigue, dizziness, insomnia, headache, hypersensitivity	Stage 4/5 CKD (GFR of less than 30 ml/min) Severe hepatic impairment	Hypersensitivity

### Tocilizumab

#### Mechanism of action

Tocilizumab is a humanized monoclonal antibody targeted against the interleukin-6 (Il-6) receptor. It competes with Il-6 and prevents its binding to its receptor. The medication was developed originally to treat rheumatoid arthritis (RA) that is refractory to conventional therapies [87]. The fostering of this medication was based on the pathologic role exerted by Il-6 and the massive and unregulated expression and production of Il-6 in patients with autoimmune diseases such as RA and Still’s disease [87, 88]. Il-6 is a pro-inflammatory cytokine secreted by numerous inflammatory cells including macrophages, B cells, and T cells among others [87]. It exerts some anti-inflammatory effects as well [87, 89]. It acts as an inhibitor of pro-inflammatory cytokines such as Il-1 and TNF-α and upregulates the expression of anti-inflammatory cytokines such as Il-1 receptor antagonist and Il-10 [90]. However, in the setting of systemic autoimmune and infectious diseases, Il-6 is a crucial mediator of inflammation and tissue destruction [88, 91]. It potentiates the B lymphocytes’ activity and stimulates the differentiation of naïve T helper cells into Th2 and Th17 cells [88, 92]. Similarly, it enhances the expression of acute phase reactants such as C-reactive protein, fibrinogen, and hepcidin. It induces the differentiation of megakaryocytes into mature platelets [88]. Hence, massive levels of Il-6 are associated with increased levels of antibodies and inflammatory molecules, as well as significant coagulopathic and thrombotic events.

#### Pharmacology

The elimination half-life of tocilizumab depends on the number of administered doses. An estimated half-life of 240 h was noted after three doses of 8 mg/kg [87]. Its volume of distribution is estimated at 6.4 L in patients with RA [93]. No evidence regarding its plasma protein binding and route of elimination is available. However, in one study, tocilizumab was evaluated in patients with RA and concurrent renal insufficiency. It was well-tolerated, and no significant side effects were reported [94]. Nonetheless, it was not assessed in patients with a glomerular filtration rate of less than 30 ml/min. Similarly, its use is not advised in patients with acute or chronic liver diseases [95].

#### Clinical evidence

The addition of tocilizumab to the treatment of severe COVID-19 patients has emanated from its well-known immunomodulatory properties. Interestingly, owing to its anti-Il-6 effect, tocilizumab can alleviate the hyperinflammatory phase of COVID-19. It can downregulate the cytokine storm syndrome that often precipitates to critical and complicated COVID-19. The use of this medication in treating COVID-19 patients has been examined in multiple studies, including multicenter observational studies and randomized clinical trials.

First, Biran et al. have conducted a multicenter retrospective observational study involving ICU patients with severe COVID-19 [96]. Out of 764 patients admitted to 13 hospitals, 27% were treated with tocilizumab in addition to standard care. The clinical outcomes encountered in those treated with tocilizumab were compared to patients receiving other medical regimens. A significant improvement in clinical outcomes and mortality was noted in the tocilizumab-treated group [96]. However, the study is subjected to the inherent biases associated with observational studies, including misclassification and selection biases. No randomization and adequate control were applied. The indications for tocilizumab administration were not clearly stated. Furthermore, no pediatric patients were enrolled in this study.

Similarly, favorable outcomes were reported in numerous observational studies performed in different parts of the world [97–102]. However, contradictory unfavorable results were conveyed by higher-quality randomized clinical trials [103, 104]. Hermine et al. have compared the efficacy of tocilizumab to usual care in a cohort of adult hospitalized patients with moderate to severe disease [103]. One hundred and thirty-one patients were enrolled in this RCT, and almost half the patients were treated with tocilizumab. No significant improvement in clinical outcomes or mortality rate was achieved by the addition of tocilizumab to usual care [103]. In a second RCT, Salvarani et al. have assessed the efficacy of tocilizumab and standard care in preventing disease progression in a cohort of 126 COVID-19 hospitalized patients with pneumonia [104]. Around 50% of the patients were randomly assigned to the tocilizumab group. No added benefit was associated with the use of tocilizumab in these patients [104]. Only adult patients were included in these two studies, and no ICU patients were enrolled in any of these studies. Similarly, both studies were limited by the small number of enrolled patients. Hence, larger RCTs involving patients from different age groups and with distinct stages of COVID-19 are needed to adequately assess the role of tocilizumab in reducing the economic, medical, and social burdens of COVID-19.

### Others: azithromycin, oseltamivir, monoclonal antibodies, vitamins and minerals

#### Azithromycin

**Fig. 2:**
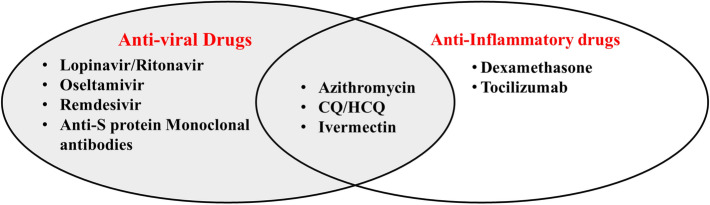
The distribution of the discussed COVID-19 therapeutics as per their main mechanism of action. Lopinavir/ritonavir, oseltamivir, remdesivir, and anti-SARS-CoV-2 spike (S) protein monoclonal antibodies are anti-viral drugs with distinct SARS-CoV-2 inhibitory effects. Azithromycin, chloroquine/hydroxychloroquine, and ivermectin display both antiviral and anti-inflammatory properties. Dexamethasone and tocilizumab display no direct antiviral properties; however, they exert anti-inflammatory effects that contribute in attenuating excessive pro-inflammatory responses

Azithromycin is a macrolide antibiotic used in the treatment of numerous bacterial illnesses including community-acquired bacterial pneumonia. It covers a broad spectrum of bacteria and is effective against numerous Gram-negative, Gram-positive, and atypical bacteria [105]. It exerts its antibacterial effect by binding to bacterial ribosome and inhibition mRNA translation and protein synthesis. It is a bacteriostatic antibiotic that leads to thwarted bacterial growth [105]. Similarly, azithromycin is known to exert antiviral and antiparasitic properties. It has been used in treating parasitic illnesses such as babesiosis, malaria, and toxoplasmosis, and viral infections such as MERS-CoV, respiratory syncytial virus, SARS-CoV-1, and lately SARS-CoV-2 [106–112]. Its anti-SARS-CoV-2 effects are mediated by azithromycin’s activity against viral entry and pro-inflammatory cytokines’ release [113]. Hence, it exerts both antiviral and anti-inflammatory effects (see Fig. 2).

To date, no RCT has endorsed the use of azithromycin in treating COVID-19 patients. This medication’s off-label use is founded on its above-mentioned antiviral properties and on evidence deriving from previous coronavirus outbreaks and low-quality observational studies [42–44]. However, as per data reported by two multicenter RCTs, a combination of HCQ and azithromycin is ineffective in treating and preventing COVID-19 progression [114, 115]. Nonetheless, only adult patients were enrolled in these studies. Furthermore, no trials have studied the efficacy of monotherapy azithromycin in treating COVID-19. A few large multicenter randomized controlled trials tackling this use are still ongoing [116, 117].

#### Oseltamivir

Oseltamivir is an anti-influenza A and B medication used in patients aged 1 year and above. It acts as a selective inhibitor of the influenza virus’s neuraminidase enzymes that are essential for viral entry, internalization, replication, and progeny release [118–121]. The drug is administered orally at a dose of 75 mg twice daily in adults and children more than 13 years and weighing more than 40 kg. In younger patients, the dosage varies depending on the weight of the patient [122]. The dosage of oseltamivir should be adjusted in patients with a GFR of less than 30 ml/min. However, oseltamivir’s use is not recommended in patients with a GFR below 10 ml/min [118]. There is no indication for dosage adjustment in patients with mild to moderate hepatic impairment. Oseltamivir is known to exhibit a low profile of drug–drug interactions [118]. Hence, it can be safely administered to a wide spectrum of patients including children and adults with mild–moderate hepatic and renal impairments.

No RCT has examined the efficacy of oseltamivir in treating adult or pediatric COVID-19 patients. Nonetheless, its off-label use has been documented in these patients. Indeed, in a large multicenter cohort study, including 582 pediatric patients from 25 European countries, oseltamivir was the fourth most-used medication following hydroxychloroquine, remdesivir, and lopinavir–ritonavir, respectively [39]. This use is based on the antiviral properties of the medication and on experience with former coronaviruses. For instance, in a retrospective study involving pediatric patients with non-SARS-CoV-2 coronaviruses treated at 15 European countries between 2016 and 2018, a faster recovery was associated with the use of oseltamivir [123]. Extensive multicenter studies are needed to recommend the off-label use of this medication in pediatric COVID-19 patients.

#### Monoclonal antibodies: bamlanivimab, casirivimab/imdevimab

On the 9th of November 2020, the USFDA has authorized bamlanivimab in adult and pediatric non-hospitalized COVID-19 patients with mild to moderate symptoms [124]. Bamlanivimab, or LY-CoV555, is a novel monoclonal antibody targeted against the spike protein of SARS-CoV-2. It hinders viral attachment to human cells and impedes its entry and replication within these cells [125]. This medication is authorized only in patients aged 12 years and above, and weighing at least 40 kg. Bamlanivimab’s use is advised in at high-risk patients age more than 65 years or having at least one of the following comorbidities: obesity (BMI ≥ 35 kg/m^2^), chronic kidney disease, diabetes, and immunosuppressive diseases. It is also indicated in patients with respiratory and cardiovascular diseases who are aged 55 years and above [126]. As for pediatric patients, its use is recommended in all at high-risk children aged at least 12 years. Children with cardiac, respiratory, neurodegenerative, and sickle cell diseases as well as obese children with a BMI of 85^th^ percentile and above are candidates for bamlanivimab [126]. Nonetheless, adult and pediatric patients requiring oxygen or respiratory support should not receive this medication [125].

Bamlanivimab’s USFDA emergency use authorization (EUA) is based on preliminary results reported to the USFDA by a couple of ongoing studies [125]. Serious safety concerns have been associated with the administration of bamlanivimab to hospitalized patients. Hence, studies exploring bamlanivimab’s use in this population has been suspended a week before the EUA’s release [125]. However, reduced rates of hospitalization and progression to severe disease have been associated with the addition of this medication to the supportive standard of care (SOC) of non-hospitalized patients [125].

**Fig. 3:**
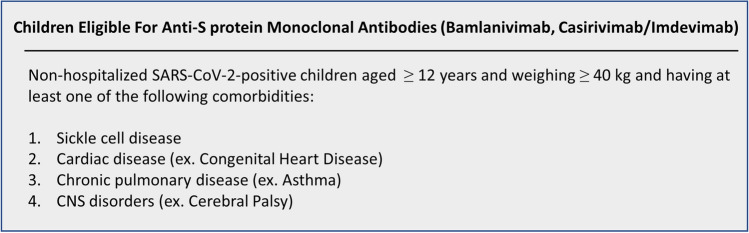
Indications for anti-SARS-CoV-2 spike protein monoclonal antibodies in children [126, 128]

Similarly, on November 21, the emergency use of a novel preparation consisting of two monoclonal antibodies, casirivimab/imdevimab (or REGN-CoV2), has been authorized by the USFDA in non-hospitalized COVID-19 patients aged 12 years and above and at high-risk for progression to severe and complicated disease (see Fig. 3) [127, 128]. Just like bamlanivimab, these antibodies are directed against the spike protein of SARS-CoV-2. They inhibit viral entry and thus replication. As per the interim results of a single clinical trial, the addition of REGN-CoV2 to SOC is associated with hampered progression of COVID-19 and diminished rates of hospitalization [129]. Nonetheless, this preparation is contraindicated in patients with respiratory distress requiring oxygen or ventilation support, and also in all hospitalized patients [129].

To note, an EUA permits the use of an unapproved medication in particular populations and supports its evaluation in clinical trials. This authorization is not equivalent to a USFDA approval of a relatively well-studied medication. An EUA is only followed by official approval if the medication is deemed effective in clinical trials.

#### Vitamins and minerals

Dietary supplements, such as vitamins C, D, and zinc, have gained increased attention over the last year. They are regarded as potential immune boosters that may induce therapeutic and prophylactic anti-COVID-19 properties. The use of these supplements is based on their well-known nutritional and immunomodulatory properties [19]. However, high-quality randomized controlled trials are needed to evaluate these supplements’ role and recommend their use in treating and preventing COVID-19.

## The levels of the reported evidence and the classes of the suggested recommendations

The evidence mentioned above is drawn from multicenter adult RCTs and international guidelines suggested by pediatric specialists. However, no pediatric patients, aged 18 years and less, have been enrolled in any of these studies. Subsequently, we postulate that the current evidence deduced from non-pediatric studies and applied to pediatric population is low-quality level C evidence (Table 1). As for adults, we suggest that most of the available evidence, inferred from numerous multicenter RCTs, is considered high-quality level A evidence. Consequently, we suggest that further pediatric studies are urgently needed to determine the most efficacious anti-SARS-CoV-2 medication that can be safely administered to these patients.

Regarding the classes of the suggested recommendations, we classified the medications as per the class of recommendation (Table 1). Indicated/recommended medications are considered class I recommendations. Medications that should or may be considered are class IIa and IIb recommendations, respectively. Unrecommended medications are considered class III recommendations. According to the USFDA, remdesivir is indicated/recommended in hospitalized children aged 12 years and above, and weighing at least 40 kg. Additionally, the USFDA has authorized the emergency use of bamlanivimab and casirivimab/imdevimab to non-hospitalized COVID-19 patients aged 12 years and above and weighing at least 40 kg who are at high-risk for complicated disease. As mentioned previously, these monoclonal antibodies are only recommended in non-hospitalized patients requiring no respiratory support, since it may prevent the progression from mild and moderate to severe and critical COVID-19 [127]. Other medications, such as hydroxychloroquine, azithromycin, tocilizumab, and ivermectin among others (see Table 1), can be occasionally considered in non-remdesivir candidates. Furthermore, as per EMA, both remdesivir and dexamethasone are recommended [130].

**Fig. 4:**
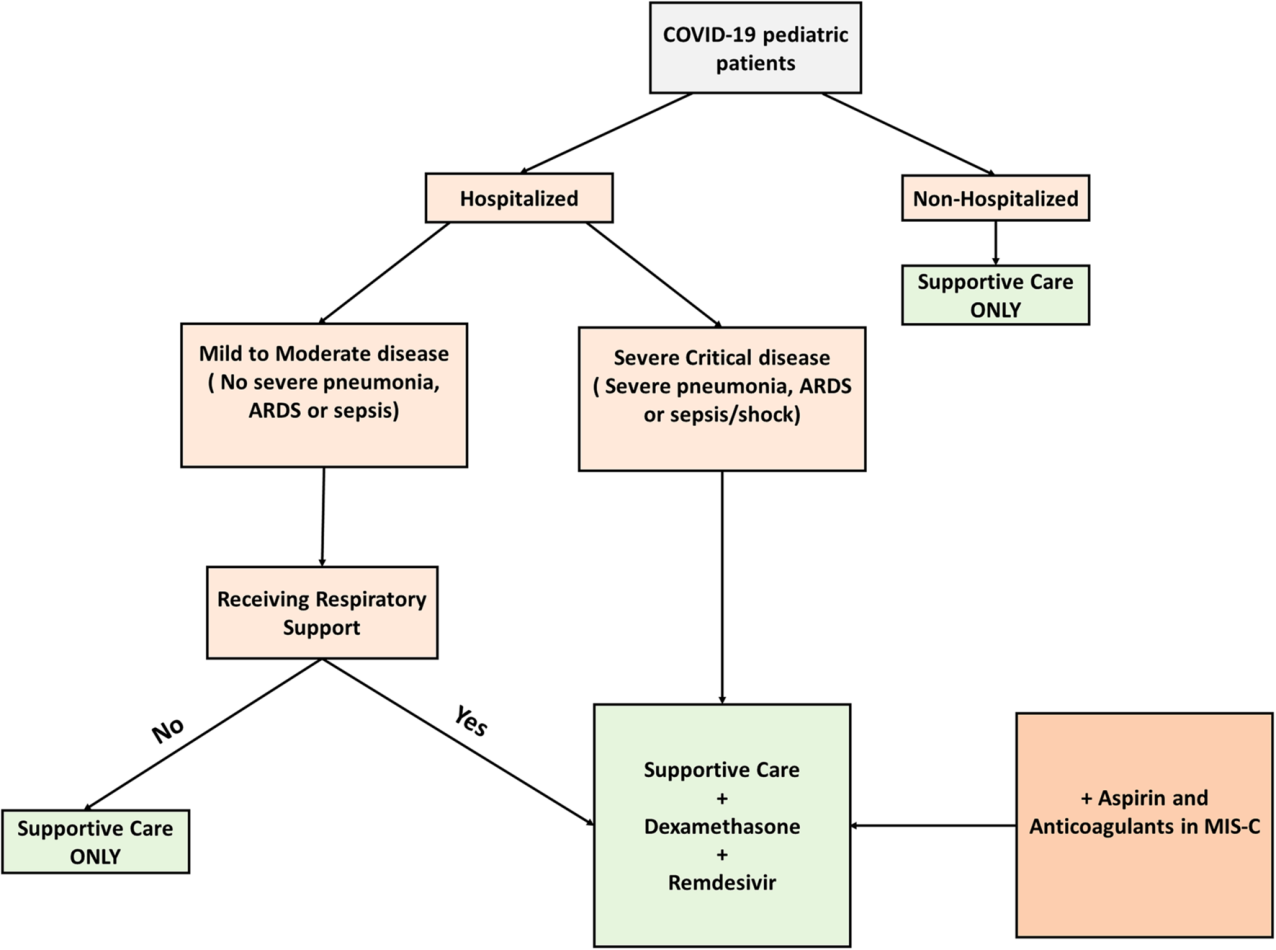
Suggested treatment algorithm. Supportive care consisting of fluids replacement and fever reduction should be offered to all COVID-19 pediatric patients regardless of hospitalization status and disease severity. Patients requiring respiratory support (oxygen or mechanical ventilation) should receive dexamethasone and remdesivir in addition to supportive care. Aspirin and anticoagulants should be add to the treatment of children with multisystem inflammatory syndrome. If remdesivir is contraindicated, other immunomodulators, such as IVIG and tocilizumab, may be considered in children with MIS as well. *ARDS* Acute respiratory distress syndrome, *MIS-C* multisystem inflammatory syndrome in children

Based on the information displayed above, we suggest the adoption of the treatment algorithm depicted in Fig. 4. Supportive care, consisting of fluids supplementation and antipyretics, should be offered to all patients regardless of the disease severity. Dexamethasone is indicated in patients with respiratory distress necessitating oxygen or ventilation support. Remdesivir should be provided to all eligible candidates requiring respiratory support. If remdesivir is contraindicated, tocilizumab may be added to treat patients with severe and complicated COVID-19 entailing excessive release of pro-inflammatory cytokines. In short, the addition of tocilizumab to the medical regimen is favored particularly in pediatric patients with MIS-C. These patients should likely receive antiplatelet medications such as aspirin and anticoagulant medications such as enoxaparin that can prevent the occurrence of life-threatening thromboembolic events. The USFDA-authorized monoclonal antibodies are indicated in non-hospitalized patients at high risk for disease progression, as indicated previously.

## Conclusion

In sum, no RCTs have included pediatric patients, to date. The bulk of the current evidence is derived and extrapolated from adult-targeted studies. Of the aforementioned medications, remdesivir is the only USFDA-approved medication in hospitalized patients. In November 2020, the USFDA has permitted the use of novel monoclonal antibodies, bamlanivimab and casirivimab/imdevimab, in non-hospitalized at-risk COVID-19 patients with mild to moderate disease necessitating no respiratory support. However, the efficacy of these preparations should be validated by high-quality clinical trials. Furthermore, only remdesivir and dexamethasone were proven to be effective in multicenter placebo-controlled RCTs, unlike the remaining medications. Nonetheless, dexamethasone is recommended by both the US National Institute of Health (NIH) and the EMA only for patients requiring respiratory support. Subsequently, its use is only endorsed in adult and pediatric COVID-19 patients maintained on oxygen therapy or mechanical ventilation. Even with all the exerted efforts and the accomplished achievements, further high-quality RCTs involving pediatric patients are urgently needed to attain top evidence and provide the highest-quality care to these patients.

Finally, vaccination remains the ultimate prevention method that may control the spread of COVID-19 and reduce its fatality. Interestingly, numerous vaccine candidates are currently studied in pre-clinical and clinical trials [131]. Nonetheless, the development of an effective anti-SARS-CoV-2 vaccine is hampered by several challenges reflected primarily by the novelty of SARS-CoV-2, the scarcity of sufficient time, and the ambiguity of the immune response mounted against SARS-CoV-2. Hence, further clinical trials are likely needed to expedite the development of these vaccines.

## Take home messages

- Little is known about the treatment of pediatric COVID-19 patients.
- The bulk of the available evidence is obtained from adult studies.
- Supportive measures directed toward controlling body temperature, preventing dehydration, and maintaining adequate oxygenation are the mainstay of COVID-19 treatment.
- Remdesivir is indicated exclusively in hospitalized patients who are aged 12 years and above.
- Dexamethasone is recommended in patients requiring respiratory support. It improves mortality in those receiving oxygen or ventilatory support.

## Abbreviations

SARS-CoV-2: Severe acute respiratory syndrome coronavirus 2
COVID-19: Coronavirus disease 2019
RCT: Randomized controlled trials
WHO: World Health Organization
USFDA: US Food and Drug Administration
SOC: Standard of care
ARDS: Acute respiratory distress syndrome
MIS-C: Multisystem inflammatory syndrome in children
CQ: Chloroquine
HCQ: Hydroxychloroquine
GFR: Glomerular filtration rate
Il1-α: Interleukin 1 alpha
TNF-α: Tumor necrosis factor alpha
Il-6: Interleukin 6
IVIG: Intravenous immunoglobulin
RdRp: RNA-dependent RNA polymerase
ICU: Intensive care unit
SARS-CoV: Severe acute respiratory syndrome coronavirus
MERS-CoV: Middle East respiratory syndrome coronavirus
S protein: Spike protein
nsp12: Non-structural protein 12
ExoN: Exoribonuclease
LY-CoV555: Bamlanivimab
REGN-CoV2: Casirivimab/imdevimab
EMA: European Medicines Agency
NIH: National Institutes of Health
EUA: Emergency Use Authorization
